# Type II Fusarium head blight susceptibility conferred by a region on wheat chromosome 4D

**DOI:** 10.1093/jxb/eraa226

**Published:** 2020-05-30

**Authors:** Benjamin Hales, Andrew Steed, Vincenzo Giovannelli, Christopher Burt, Marc Lemmens, Marta Molnár-Láng, Paul Nicholson

**Affiliations:** 1 Department of Crop Genetics, John Innes Centre, Norwich Research Park, Norwich, UK; 2 University of Natural Resources and Life Sciences, Institute for Biotechnology in Plant Production, Department of Agrobiotechnology, IFA Tulln, Tulln, Austria; 3 Agricultural Institute, Centre for Agricultural Research, Martonvásár, Hungary; 4 MPI of Molecular Plant Physiology, Germany

**Keywords:** Aneuploid, barley, deletion, Fusarium, scab, susceptibility, wheat

## Abstract

Fusarium head blight (FHB) causes significant grain yield and quality reductions in wheat and barley. Most wheat varieties are incapable of preventing FHB spread through the rachis, but disease is typically limited to individually infected spikelets in barley. We point-inoculated wheat lines possessing barley chromosome introgressions to test whether FHB resistance could be observed in a wheat genetic background. The most striking differential was between 4H(4D) substitution and 4H addition lines. The 4H addition line was similarly susceptible to the wheat parent, but the 4H(4D) substitution line was highly resistant, which suggests that there is an FHB susceptibility factor on wheat chromosome 4D. Point inoculation of Chinese Spring 4D ditelosomic lines demonstrated that removing 4DS results in high FHB resistance. We genotyped four Chinese Spring 4DS terminal deletion lines to better characterize the deletions in each line. FHB phenotyping indicated that lines del4DS-2 and del4DS-4, containing smaller deletions, were susceptible and had retained the susceptibility factor. Lines del4DS-3 and del4DS-1 contain larger deletions and were both significantly more resistant, and hence had presumably lost the susceptibility factor. Combining the genotyping and phenotyping results allowed us to refine the susceptibility factor to a 31.7 Mbp interval on 4DS.

## Teaser

We have identified a 31.7 Mbp interval on the short arm of wheat chromosome 4D that is involved in increased Type II susceptibility to Fusarium head blight.

## Introduction

Fusarium head blight (FHB) is an economically important fungal disease of various cereal crop species, in particular wheat (*Triticum aestivum*) and barley (*Hordeum vulgare*). In wheat, the primary symptom is the premature bleaching of spikelets that progressively spreads through the head. Infected spikelets produce shrivelled and chalky grain, which can have a significant negative impact on yield. Furthermore, mycotoxins accumulate in infected grain, which are harmful to humans and animal consumers. The most important mycotoxin is deoxynivalenol (DON) which acts as a virulence factor in wheat by promoting the spread of the fungus (Bai *et al.*, 2002; Langevin *et al.*, 2004). *Fusarium graminearum* and *F. culmorum* are the most prevalent species responsible for FHB. Both species are capable of producing large quantities of DON (Scherm *et al.*, 2013) and hence tend to be the most aggressive pathogens of wheat.

Resistance to initial infection (Type I) and to the spread of infection through the rachis (Type II) were first proposed by Schroeder and Christensen (1963) and remain the two most widely considered forms of resistance. Numerous small-effect Type II and fewer Type I FHB quantitative trait loci (QTLs) have been reported and are reviewed by Buerstmayr *et al.* (2009) and more recently by Buerstmayr *et al.* (2019). In addition to these two main types of FHB resistance, there is resistance to kernel infection (Type III), host tolerance to FHB and/or DON (Type IV), and resistance to the accumulation of DON (Type V) (Boutigny *et al.*, 2008; Gunupuru *et al.*, 2017). Single amino acid changes to the DON target, ribosomal protein L3 (RPL3), have been demonstrated to improve tolerance to DON in yeast, and hence this is a possible target to improve type IV resistance (Mitterbauer *et al.*, 2004; Lucyshyn *et al.*, 2007). Type V resistance is commonly considered to be a component of Type II resistance, as it typically limits disease spread (Gunupuru *et al.*, 2017), and can be subdivided into Class 1, processes that chemically modify DON to a less toxic form; and Class 2, processes that prevent the accumulation of DON and other trichothecene mycotoxins (Boutigny *et al.*, 2008). The most widely reported form of host detoxification of DON is by UDP-glucosyltransferase (UGT) proteins, which glucosylate DON to the less toxic DON-3-*O*-glucoside (D3G) (Poppenberger *et al.*, 2003). More recent studies have identified other pathways capable of detoxifying DON. For example, bacterial aldo-keto reductases were demonstrated to be involved in epimerizing DON to 3-*epi*-DON (Hassan *et al.*, 2017; He *et al.*, 2017).

Wheat and barley differ noticeably in Type II resistance. Wheat typically possesses some degree of Type II susceptibility whilst, in contrast, barley is generally highly resistant to fungal spread through the rachis (Langevin *et al.*, 2004). Furthermore, whilst DON has been shown to function as a virulence factor in wheat (Langevin *et al.*, 2004), DON does not appear to possess such a role during infection of barley heads (Maier *et al.*, 2006).

The reasons for this marked difference in Type II susceptibility of wheat and barley are not well understood. Defined genetic stocks of wheat containing all or part of barley chromosomes offer an insight into which barley chromosomes contribute most strongly to Type II FHB resistance and whether this resistance can be expressed, and potentially utilized, in a wheat genetic background. Herein, we report on a series of experiments to establish whether this difference in FHB susceptibility is because barley carries genes conferring resistance, wheat carries genes conferring susceptibility, or whether it is a combination of both factors. Following this, we investigated the location of a major effect identified on wheat chromosome 4D that appears to significantly compromise resistance to FHB spread through the rachis (Type II resistance).

To date, there have been few reports of FHB susceptibility factors. Garvin *et al.* (2015) identified a spontaneous deletion of a portion of the long arm of 3D, which appeared to be responsible for increased FHB resistance, suggesting that the deleted region carries an FHB susceptibility factor in the cultivar Apogee. Ma *et al.* (2006) point-inoculated the existing ditelosomic (DT) lines of Chinese Spring that each lack individual chromosome arms. They found that the loss of individual chromosome arms can improve, as well as compromise, FHB resistance (Ma *et al.*, 2006). Their data suggested that some chromosome arms, especially 7AS, 3BL, 7BS, and 4DS, are likely to contain FHB susceptibility factors (Ma *et al.*, 2006). Although the gene(s) underlying *Fhb1*, the most widely deployed FHB resistance QTL, remains controversial, there is evidence that *Fhb1* may be considered a disrupted susceptibility factor (Su *et al.*, 2018, 2019). Plant hormones play an important role in responding to disease. Host response to FHB infection is particularly sensitive to disrupting phytohormone production or perception. Plants insensitive to ethylene and brassinosteroid signalling exhibit increased FHB resistance, which suggests that the fungus is exploiting such physiological processes (Chen *et al.*, 2009; Goddard *et al.*, 2014). There is significant potential in identifying and characterizing susceptibility factors, with the aim of eliminating them from elite cultivars to enhance resistance to FHB and other economically important diseases.

## Materials and methods

### Plant material

Wheat–barley addition, substitution, and translocation lines were developed at the Hungarian Academy of Sciences, Agricultural Institute, Centre for Agricultural Research, Hungary (Table 1). An independent set of wheat–barley addition lines, of the wheat variety Chinese Spring and the barley donor variety Betzes, were generated by Islam *et al.* (1981) and obtained from the Genetic Resources Unit at the John Innes Centre, Norwich, UK.

**Table 1.** Wheat–barley addition, substitution, translocation, and centric fusion lines used in FHB experiments

Line abbreviation	Description	Reference
Mv9kr1	Martonvasari9 *kr1*	Molnar-Lang *et al.* (1996)
1HS add	Mv9kr1–Igri 1HS disomic addition	Szakacs and Molnar-Lang (2007)
2H add	Mv9kr1–Igri 2H disomic addition	Szakacs and Molnar-Lang (2007)
3H add	Mv9kr1–Igri 3H disomic addition	Szakacs and Molnar-Lang (2007)
4H add	Mv9kr1–Igri 4H disomic addition	Szakacs and Molnar-Lang (2007)
6HS add	Mv9kr1–Igri 6HS disomic addition	Szakacs and Molnar-Lang (2010)
7H add	Mv9kr1–Igri 7H disomic addition	Szakacs and Molnar-Lang (2010)
2D-1H trans	2DS.2DL-1HS translocation	Nagy *et al.* (2002)
3HS.3BL centric	3HS.3BL centric fusion	Nagy *et al.* (2002)
4H(4D) sub	4H(4D) wheat–barley substitution	Molnar *et al.* (2007)
6B-4H trans	6BS.6BL–4HL translocation	Nagy *et al.* (2002)
7D-5H trans	5HS-7DS.7DL wheat–barley translocation	Kruppa *et al.* (2013)

The primary wheat parent was Martonvasari 9 kr1 (Mv9kr1) for all lines, and the barley donor parents were Igri or Betzes. Associated references contain detailed descriptions of line generation and composition.

Chinese Spring and its 4D DT lines were acquired from the Germplasm Resource Unit, John Innes Centre, Norwich, UK. The lines DT(4DL) and DT(4DS) lack 4DS and 4DL, respectively. Four homozygous Chinese Spring terminal deletion (CSTD) lines of 4DS, described by Endo and Gill (1996), were obtained from Kansas State University, USA. The lines acquired were 4532 L1 [fraction length (FL)= 0.53], 4532 L2 (FL=0.82), 4532 L3 (FL=0.67), and 4532 L4 (FL=0.77), henceforth referred to as del4DS-1, del4DS-2, del4DS-3, and del4DS-4, respectively.

### Marker development and genotyping

Homoeologue non-specific markers were designed to simultaneously amplify fragments of homoeologous genes on 4A, 4B, and 4D. Sequence information of 4D genes and corresponding homoeologous genes was obtained from Ensembl Plants (http://plants.ensembl.org/Triticum_aestivum/Info/Index). Gene names and the physical positions reported correspond to the IWGSC RefSeq v1.1 wheat genome assembly (IWGSC, 2018). Sequence insertions and deletions (indels) between homoeologous gene sequences were exploited to enable distinction of the three resulting PCR products. Forward primers were M13-tailed to enable incorporation of a fluorescent adaptor to PCR products, as described by Schuelke (2000). A total of 37 markers designed as such were used to characterize the deletions in four 4DS CSTD lines (Table 2).

**Table 2.** Homoeologue non-specific markers used to genotype four Chinese Spring 4DS terminal deletion lines

Marker	Forward primer	Reverse primer	Fragment A; B; D (bp)	4D gene target
BH0001	tgtaaaacgacggccagtTCCTCCAATAAGAAGGTATGTC	TGGCACTGCCCTTATAGCAA	356; 330; 228	TraesCS4D02G001400
BH0002	tgtaaaacgacggccagtTGTCGTTGTTCCAGTTAAAG	TCAGGCGCATCAGACATTTG	205; 172; 163	TraesCS4D02G009200
BH0013	tgtaaaacgacggccagtGGGGAATTGTCCAAAGCGT	TGCAAGAGATGTTGGGATTTT	211; 155; 207	TraesCS4D02G014500
BH0003	tgtaaaacgacggccagtCTCCACTTTATCATTTGAAGACA	ACAAAACCTTTCACATGGCC	452; 264; 491	TraesCS4D02G017300
BH0004.2	tgtaaaacgacggccagtGTGTTCCCATTGTCGCCG	TAGTCCGCCTCCTTGCTCCT	168; 152; 194	TraesCS4D02G035700
BH0025.2	tgtaaaacgacggccagtACAATCCCGAGGTTGCCAGA	CGAAGAGGAGGGCATACATA	275; 359; 378	TraesCS4D02G039400
BH0005.2	tgtaaaacgacggccagtTGGTGCTTCATTATCCTTCTGAT	TGGTGTCCAGAGTAAACTCGATA	443; 448; 319	TraesCS4D02G040700
BH0020	tgtaaaacgacggccagtCGACCTCCTCTCAGCTTTTAG	ATGAGGATACACGGTGCTGC	304; 193; 220	TraesCS4D02G045500
BH0029	tgtaaaacgacggccagtGAGCAGATCTTCAACGTACG	ATCACAAAGGGATGGACCTG	183; 196; 159	TraesCS4D02G050300
BH0024	tgtaaaacgacggccagtAAAGTAAAATCCTCTTCCCTGAG	GCTAAACTTGCTGTCAGACAAG	274; 298; 389	TraesCS4D02G051400
BH0006.2	tgtaaaacgacggccagtGGCCAAGGTGCGTAATCCA	CGCGAGCTGAACACAAGC	265; 121; 313	TraesCS4D02G052300
BH0022	tgtaaaacgacggccagtAGTATTAGGCAATGTGTTCCACT	TGAGAAGGTTCCAAGAACCAAC	288; 459; 260	TraesCS4D02G057100
BH0021	tgtaaaacgacggccagtTCATTCAACATGCAGATCTAGGC	GACAAACTTCAATGGCATAAGC	123; 155; 130	TraesCS4D02G065300
BH0014	tgtaaaacgacggccagtCCATTGCATTCCTTCACTTGT	CGTCGTCCCATACTTCACAAA	110; 113; 107	TraesCS4D02G066900
BH0026	tgtaaaacgacggccagtCGATACACCAGTTAATTGAAATATG	CTAGGAGTTCCTTCATGGACATT	289; 471; 318	TraesCS4D02G073200
BH0015.2	tgtaaaacgacggccagtCACAACTTGTGCAGGTATAACC	GGAAAGTCAAGACAGGCACAA	198; 346; 426	TraesCS4D02G074200
BH0008	tgtaaaacgacggccagtGTATCGACGAAGCCGCAGTT	TTCCGGAGCGTCCTACGACAA	309; 190; 199	TraesCS4D02G074500
BH0040	tgtaaaacgacggccagtGCGCAGTGAGACAAAACTC	AAGTAGAAGAGCAGCGCCAT	442; 448; 451	TraesCS4D02G075300
BH0041	tgtaaaacgacggccagtAACAAATCCATGTGACCCC	CTACAAGGACGCGTGGTTAT	299; 338; 302	TraesCS4D02G076000
BH0042	tgtaaaacgacggccagtCGGACAACATTTCAGGATTTC	ACCGGAACAAGGCTGCAC	379; 135; 125	TraesCS4D02G077600
BH0027.3	tgtaaaacgacggccagtGGTAACATTCCTTTGGTATACTCGG	TGTGCTAAGATCTACAACATC	303; 350; 266	TraesCS4D02G078900
BH0032	tgtaaaacgacggccagtTTGTGGCCTGCTTACATTGC	TGATCTGCAGGTGTTGGC	317; 305; 300	TraesCS4D02G079900
BH0033	tgtaaaacgacggccagtTGCCCGTGTTTTATGCACTG	GGTAAGTAAAATGGGAAGAAAGC	201; 167; 185	TraesCS4D02G081000
BH0034	tgtaaaacgacggccagtCTGCCGTATCTCCAACTC	ATGAGCGCCATCAGGAAC	209; 297; 217	TraesCS4D02G082500
BH0035	tgtaaaacgacggccagtACGCGGACCCGAATTCAAA	TCCTTGGGCATAGAGGAAG	190; 167; 162	TraesCS4D02G083100
BH0036	tgtaaaacgacggccagtATGTTAGCCGTCCTTTGTTTC	TGGCTGACAGCTATACTTCTAGT	246; 255; 223	TraesCS4D02G084000
BH0037	tgtaaaacgacggccagtGACGGACAATTCTTATGATTGTG	TATGTCCTGCCCCTTCTCCAT	191; 187; 166	TraesCS4D02G085100
BH0038	tgtaaaacgacggccagtATCTGCGTCCAGGTGAGC	TCAGCTAAGACAACTGGCAC	359; 341; 318	TraesCS4D02G085900
BH0009.3	tgtaaaacgacggccagtTAGAGGGAGCAGGGATGACAT	TCTCCGTCTGGTTCATTCGT	106; 103; 111	TraesCS4D02G087200
BH0010.2	tgtaaaacgacggccagtACGTGGTCTTCAAATCTGGC	CTGCAATATAAGGTGGCAAATC	189; 155; 159	TraesCS4D02G098400
BH0017	tgtaaaacgacggccagtCAGATTGTACGAACATCTTCTGC	AGCAGAACAAAATCTCATGG	252; 246; 263	TraesCS4D02G105100
BH0018	tgtaaaacgacggccagtGTGAGCAGAGCACCCTCC	CTGCACCACCACAGAAAAGA	226; 195; 214	TraesCS4D02G107300
BH0011	tgtaaaacgacggccagtATGCTCGTCTTCATCGAGGTAA	ATGCATTGCAGACACATCAAG	128; 160; 135	TraesCS4D02G114700
BH0012.2	tgtaaaacgacggccagtGGTCCTTCATGAAGCTTGTTC	GGCAAATAAGAGAGTTGCATAGG	275; 289; 280	TraesCS4D02G117800
BH0030	tgtaaaacgacggccagtGGCAATGTGATCCTGCAGTTC	GCCCAAAGAAATAGCAAGGGAAA	145; 174; 189	TraesCS4D02G126600
BH0057	tgtaaaacgacggccagtGCACATCCTGCTGTACCA	CTCCTTGGGAATCTTAATGCA	464; 356; 322	TraesCS4D02G147800
BH0058	tgtaaaacgacggccagtCCATTTAGATTCATGGCGAT	AGGCATATTGCAAACCCAAC	190; 315; 179	TraesCS4D02G149800

Primer sequences, fragment sizes (corresponding to the 4A, 4B, and 4D homoeologous gene targets), and the 4D gene target of markers used to characterize the deletion sizes present in four Chinese Spring 4DS terminal deletion lines. The lower case sequence in the forward primer indicates the M13 tail. All markers were amplified at 58 °C annealing temperature.

DNA was extracted from freeze-dried leaf tissue as described by Pallotta *et al.* (2003). PCRs were prepared using HotStarTaq Mastermix (Qiagen) following the manufacturer’s instructions and amplified using the following steps: 95 °C 15 min; 35 cycles of 95 °C 1 min, 58 °C 1 min, 72 °C 1 min; and 72 °C 10 min. PCR products were separated using an ABI 3730xl DNA analyser (Applied Biosystems) and resolved using Peak Scanner 2 software (Applied Biosystems). Up to five markers were multiplexed following PCR to increase assay efficiency.

Primers were designed to specifically amplify within a 5H barley UGT (HORVU5Hr1G047150), whilst avoiding amplification of wheat orthologues (primer sequences: GATGAGGTTTGAGATTTGCGGA, CACGAGCACAACAGATGAATTCA). PCRs were prepared using Taq Mastermix (Qiagen) and amplified using the following PCR settings: 94 °C 3 min; 35 cycles of 94 °C 30 s, 58 °C 30 s, 72 °C 1 min; and 72 °C 10 min. PCR products were separated on a 0.8% (w/v) agarose gel.

### FHB evaluation and statistical analysis

Highly virulent DON-producing isolates of *F. graminearum* (UK1) or *F. culmorum* (Fu42) were used in disease experiments. Production of inoculum was carried out as described previously in Gosman *et al.* (2005). Wheat heads were inoculated at mid-anthesis. The conidial suspension, adjusted to 1×10^6^ spores ml^−1^, was injected into a spikelet approximately central on the wheat head. The spread of disease symptoms was scored at 2–4 d intervals between 7 and 21 days post-inoculation (dpi). Polytunnel experiments were organized in a randomized complete block design with four replicates each containing four or five plants per line. For the glasshouse experiment, at least 16 plants per lines were randomized and individual inoculated heads were considered as replicates.

Disease data were analysed using a linear mixed model (REML) in Genstat software (v18.1) to assess the variation attributable to line (fixed), inoculation date (fixed), the interaction between line and inoculation date (fixed), and replicate (random), where factors were significant in the model. Data from which residuals were not normally distributed or where residuals did not appear independent of fitted values were log10 transformed, which was sufficient in correcting for these assumptions. Predicted mean and SE values were calculated for lines included in the REML. Pairwise comparisons were made between the wild-type wheat parent/genetic background and the other genotypes tested in each experiment using Fisher’s protected least significant difference. All predicted values generated from transformed data were back-transformed to the original scale for presentation.

### DON evaluation and statistical analysis

DON was purified to >98% at IFA-Tulln, as described by Altpeter and Posselt (1994). DON application was carried out on wheat spikes at mid-anthesis, following a protocol modified from Lemmens *et al.* (2005). Two adjacent spikelets opposite to each other on the wheat head and approximately central on the head were cut with scissors approximately central on the spikelet. At 1–2 h after cutting, 10 µl of DON solution [10 mg ml^–1^ amended with 0.01% (v/v) Tween-20] was applied to the two outer florets of each cut spikelet, between the palea and lemma. To increase the humidity at the site of DON application, treated wheat heads were bagged. At 48 h post-application, the DON application was repeated, and heads were bagged again. Hence, each treated wheat head received a total application of 0.8 mg of DON. After a further 48 h, crossing bags were removed from the DON-treated heads. The severity of bleaching for each treated wheat head was scored, out of 10, daily between 5 d and 9 d post-application (from the first application). A score of zero was given when no evidence of DON damage was present and a score of 10 was recorded when the spike was completely bleached above the point of DON application. Scores between 1 and 9 were used to record the progressive yellowing and bleaching of the DON-treated wheat heads, which occurred relatively uniformly above the point of DON application in the case of Chinese Spring (Supplementary Fig. S1 at *JXB* online). After the experiment, DON-treated and untreated heads from each plant were harvested. From each plant with a DON-treated head, a comparable untreated head (with similar spikelet number and head length) was selected for grain weight analysis. Grain number and grain weight data were collected from DON-treated and comparable untreated heads from each plant, to observe any difference in the effect of DON on grain filling.

DON bleaching data and associated grain data were analysed using a REML. In all cases of both DON bleaching data and grain data, transformation of data was not necessary, as residuals appeared to be normally distributed and independent of fitted values based on visual checking. For bleaching data, line was included as a fixed term and replicate as a random term in the model. For DON grain data, the REML model was constructed using line, treatment (DON-treated or untreated heads), and the interaction between line and treatment as fixed terms, and replicate as a random term.

## Results

### Effect of barley chromosome additions, substitutions, translocations, and centric fusions on Type II FHB susceptibility in the winter wheat variety Martonvasari 9 (Mv9kr1)

**Fig. 1.**
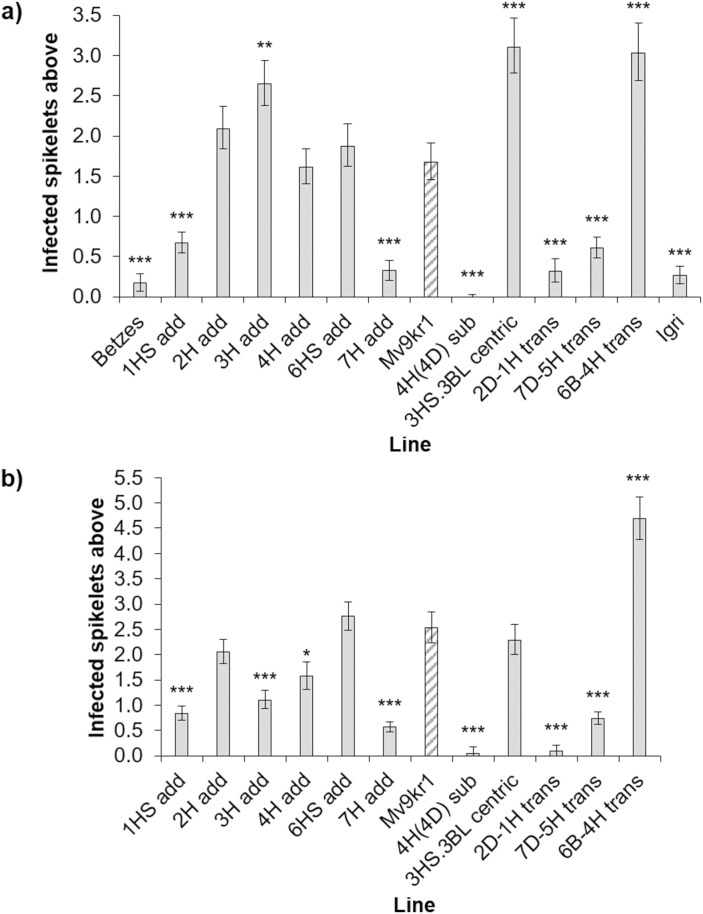
FHB disease above the inoculation point in wheat–barley addition, substitution, translocation, and centric fusion lines from (a) polytunnel experiment 1, including barley parents Igri and Betzes as controls, and (b) polytunnel experiment 2. Predicted means were generated using a linear mixed model. Error bars are ±SE. *P<0.05; **P< 0.01; ***P<0.001 compared with Mv9kr1.

FHB point inoculation experiments of the wheat–barley material were conducted twice and are described as experiment 1 (Fig. 1a) and experiment 2 (Fig. 1b) henceforth. The experiments showed very similar results for most of the lines tested. FHB symptoms were always restricted in both barley varieties, Igri and Betzes, and did not spread from the inoculated spikelet. For this reason, Igri and Betzes were only included as control lines in experiment 1 (Fig. 1a). The primary wheat parent, Mv9kr1, was susceptible to the spread of the fungus in both repeats of the experiment.

The addition of barley chromosomes 2H (2H add) and 6HS (6HS add) appeared to have no effect on FHB resistance in either experiment. Disease symptoms in these lines were not statistically significantly different from those of Mv9kr1. The 6BS.6BL-4HL translocation (6B-4H trans) was significantly more susceptible than Mv9kr1 (*P* <0.001 in both experiments). Whilst the 3HS.3BL centric fusion line (3HS.3BL centric) was more highly susceptible in experiment 1 (*P* <0.001), the line showed similar disease to Mv9kr1 in experiment 2 (*P*=0.566). The addition of chromosomes 1HS (1HS add) and 7H (7H add), in addition to the 5HS-7DS.7DL wheat–barley translocation (5H-7D trans) and the 2DS.2DL-1HS translocation line (2D-1H trans), all showed highly significant increases in FHB resistance compared with Mv9kr1 (*P* <0.001 in both experiments for all lines). The 3H addition (3H add) was inconsistent between the two experiments. In experiment 1, the 3H addition was significantly more susceptible to FHB than Mv9kr1 (*P*=0.004) whilst, in experiment 2, it was significantly more resistant (*P*<0.001).

A particularly strong resistant phenotype was seen with the 4H(4D) substitution, in which disease was almost entirely restricted to the inoculated spikelet in both experiments (*P*<0.001 in both instances). In contrast to this, the addition of barley 4H (4H add) showed similar disease levels to Mv9kr1 in experiment 1 (*P*=0.841, Fig. 1a) and exhibited only a small increase in resistance in experiment 2 (*P*=0.021, Fig. 1a).

### Effect of barley chromosome additions, substitutions, translocations, and centric fusions on Type II FHB susceptibility in the spring wheat variety Chinese Spring

**Fig 2.**
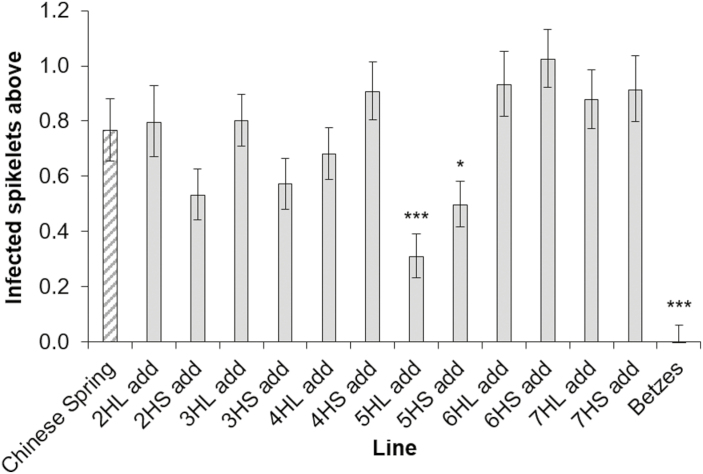
FHB disease, as a percentage of total number of bleached spikelets, from data combined from 13 dpi and 14 dpi. Predicted means were generated using a linear mixed model. Error bars are ±SE. *P=0.05–0.01; ***P<0.001 compared with Chinese Spring.

An FHB point inoculation experiment was performed on wheat–barley addition lines of the varieties Chinese Spring and Betzes, respectively (Fig. 2). These lines include addition lines of 5HS and 5HL, which were absent in the lines generated in the Mv9kr1 wheat background. As previously observed, Betzes showed almost no disease spread from the inoculation point. Chinese Spring, on the other hand, showed evidence of disease spread. FHB symptoms in the majority of addition lines were not significantly different from those in Chinese Spring.

The 5HL addition line exhibited significantly increased FHB resistance when compared with Chinese Spring (*P*<0.001), although the line was still significantly more susceptible than Betzes (*P*=0.042). The 5HS addition line was also statistically significantly more resistant compared with Chinese Spring (*P*=0.039). A marker targeting the barley UGT gene, HORVU5Hr1G047150, confirmed that this gene was present in Betzes and the 5HL addition line, but was absent in the 5HS addition line (Supplementary Fig. S2). Consistent with the previous experiments, the 4HL and 4HS addition lines both showed similar FHB susceptibility to Chinese Spring.

### Type II FHB susceptibility and DON susceptibility in Chinese Spring 4D ditelosomic lines

**Fig. 3.**
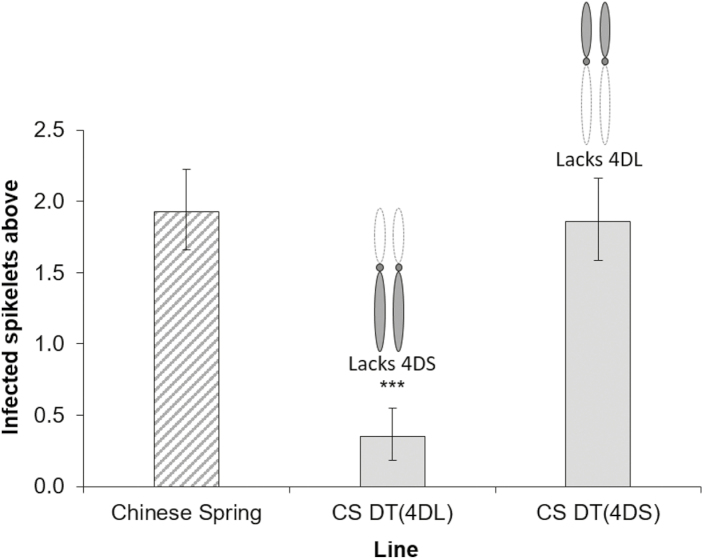
FHB disease at 17 dpi in euploid Chinese Spring and 4D ditelosomic lines DT(4DL) and DT(4DS), missing 4DS and 4DL, respectively. Diagrams of 4D are included above ditelosomic lines to illustrate their genetic state. Error bars are ±SE. ***P<0.001 compared with Chinese Spring.

The contrast in the effect of adding 4H or substituting 4D with 4H indicated that the presence of 4D may be responsible for a significant proportion of the susceptibility of both Mv9kr1 and Chinese Spring. To test this possibility, Chinese Spring and two DT lines: DT(4DL) and DT(4DS), missing 4DS and 4DL, respectively, were tested in three independent FHB point inoculation experiments. Data are presented here from a 2013 experiment conducted in a glasshouse, but the results were replicated in a 2013 experiment under controlled conditions and in a polytunnel experiment conducted in 2016. Chinese Spring and DT(4DS), missing 4DL, showed very similar disease symptoms to each other (Fig. 3). In contrast to this, DT(4DL), missing 4DS, was highly resistant to the spread of infection when compared with wild-type Chinese Spring (*P*<0.001).

**Fig. 4.**
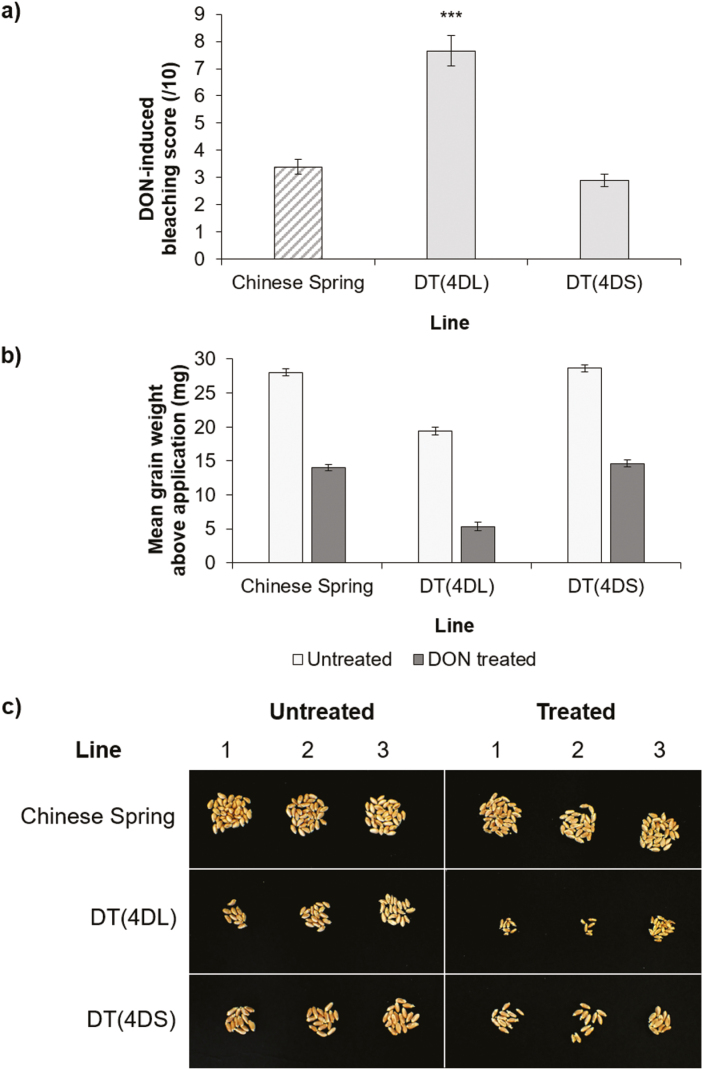
DON application experiment to heads of Chinese Spring and ditelosomic lines DT(4DL) and DT(4DS), lacking 4DS and 4DL, respectively. (a) Average DON bleaching scores at 7 d post-application. Predicted means were generated using a linear mixed model. Error bars are ±SE. *P*<0.001 compared with Chinese Spring. (b) DON-treated and untreated mean grain weights (mg) above the application point, or the comparable point in untreated heads, dissected after the experiment. Predicted means were generated using a linear mixed model. Error bars are ±SE. (c) Photograph showing three representative examples of untreated and DON-treated grain taken from above the DON application point, or the comparable point in untreated heads.

DON is widely believed to contribute towards Type II susceptibility by promoting the spread of FHB. Hence, it is possible that the susceptibility factor may be responding to DON and not the fungus itself. To confirm whether DON is involved, we applied purified DON to wheat heads of Chinese Spring and two DT lines; DT(4DL) and DT(4DS). Chinese Spring was moderately susceptible to DON, with an average bleaching score of 3.39 (Fig. 4). DT(4DS), lacking 4DL, was not significantly different from Chinese Spring (mean=2.88; *P*=0.222) (Fig. 4). On the other hand, DT(4DL), lacking 4DS, was significantly more susceptible to DON-induced bleaching (mean=7.64; *P*<0.001) (Fig. 4).

Grain was harvested and dissected from DON-treated and untreated heads to assess any difference in grain weight. These data mirrored the bleaching data. Chinese Spring and DT(4DS) showed similar reductions in grain weight when comparing DON-treated and untreated heads (percentage reductions of 50.0% and 48.9%, respectively) (Fig. 4). Average grain weight in untreated heads of DT(4DL) was smaller than in untreated Chinese Spring. However, grain of DON-treated DT(4DL) heads had a proportionally greater reduction in grain weight compared with untreated heads (72.3%) (Fig. 4). The difference is evident when visually comparing treated and untreated grain from the three lines; treated grain from DT(4DL) are visibly smaller than those of Chinese Spring and DT(4DS) (Fig. 4).

These data suggest that DON is not implicated in the function of the susceptibility factor. However, there does appear to be an independent DON resistance factor also on 4DS.

### Precise characterization of deletion sizes in Chinese Spring 4DS terminal deletion lines

Experiments using 4D DT lines strongly suggest that the FHB susceptibility attributed to chromosome 4D is isolated to the short arm (4DS). Genotyping was performed on four Chinese Spring lines with terminal deletions on 4DS to verify the deletions present and more precisely position the deletion breakpoint in each line relative to the physical map. Markers were designed that can reliably detect genes on 4D and their homoeologues on 4A and 4B. The ability to detect and distinguish all three homoeologues provides two internal positive controls for each marker when identifying deletions of any particular homoeologue. Up to five markers, tagged using different fluorophores (NED, FAM, PET, or VIC), were multiplexed into a single sample for efficiency, using markers designed to produce PCR product sizes sufficiently different for each gene target and its respective homoeologues when resolved using capillary electrophoresis (Supplementary Fig. S3).

Genotyping was successful in identifying genes and their respective physical positions, flanking the deletion breakpoint in all four 4DS CSTD lines (Table 3). A marker (BH0001) targeting the gene TraesCS4D02G001400 at the extreme distal end of 4DS confirmed that all four lines were true terminal deletions. The terminal deletion in del4DS-2 extends to between 50.6 Mbp and 51.6 Mbp. Line del4DS-4 is deleted up to between 53.9 Mbp and 54.8 Mbp. The deletion in del4DS-3 ends between 83.3 Mbp and 85.6 Mbp. The deletion breakpoint in the CSTD line containing the largest terminal deletion, del4DS-1, ends between 111.1 Mbp and 140.9 Mbp.

**Table 3.** Flanking genes and markers of deletion breakpoints in four Chinese Spring 4DS terminal deletion lines

Line	Left flank gene	Left flank marker	Right flank gene	Right flank marker	Breakpoint interval (kb)
del4DS-2	TraesCS4D02G076000	BH0041	TraesCS4D02G077600	BH0042	976
del4DS-4	TraesCS4D02G079900	BH0032	TraesCS4D02G081000	BH0033	949
del4DS-3	TraesCS4D02G105100	BH0017	TraesCS4D02G107300	BH0018	2313
del4DS-1	TraesCS4D02G126600	BH0030	TraesCS4D02G147800	BH0057	29776

The breakpoint interval is the size of the interval between two adjacent markers where the marker signal was retrieved, indicating the end of the deletion.

### Type II FHB susceptibility and DON susceptibility in Chinese Spring terminal deletion lines of 4DS

**Fig. 5.**
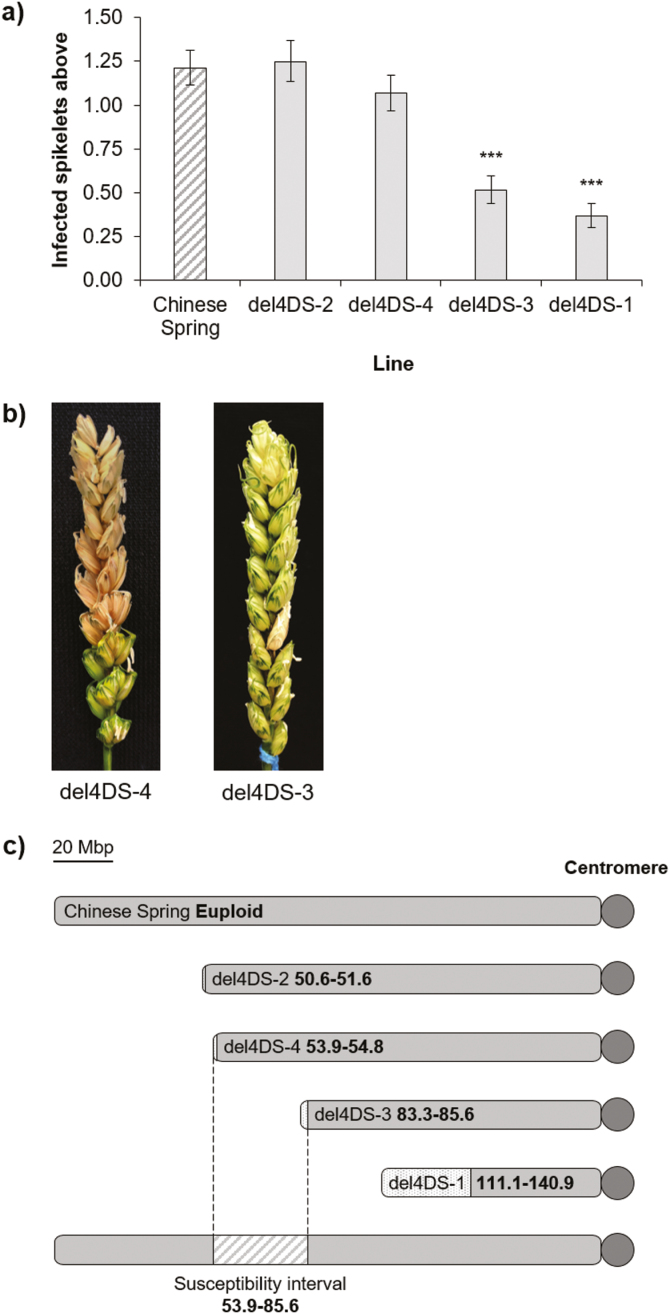
(a) FHB disease above the inoculation point at 13 dpi, following point inoculation of euploid Chinese Spring and four terminal deletion bins: del4DS-2, del4DS-4, del4DS-3, and del4DS-1. Error bars are ±SE. ***P<0.001 compared with Chinese Spring. (b) Representative FHB disease symptoms in the Chinese Spring terminal deletion lines del4DS-4 and del4DS-3 at 16 dpi. (c) Diagrams of 4DS in euploid Chinese Spring and four 4DS terminal deletion lines, as characterized by genotyping with 35 markers spanning 4DS. The spotted interval indicates the breakpoint interval; the distance between two markers where the 4D signal was retrieved. The bottom diagram indicates the interval on 4DS inferred to contain an FHB susceptibility factor (diagonal stripes), following point inoculation of the Chinese Spring terminal deletion lines. Values in bold indicate the physical position in Mbp.

Euploid Chinese Spring and the four genotyped 4DS CSTD lines (del4DS-2, del4DS-4, del4DS-3, and del4DS-1, in ascending order of terminal deletion size) were point-inoculated in a polytunnel experiment in 2017 (Fig. 5). Chinese Spring showed moderate levels of disease in this experiment, with mean disease above the inoculation point of 1.84 bleached spikelets at 13 dpi. Lines del4DS-2 (*P*=0.796) and del4DS-4 (*P*=0.278) showed similar disease levels to that of euploid Chinese Spring (Fig. 5). Lines del4DS-3 and del4DS-1 both had significantly reduced disease with respect to euploid Chinese Spring (*P*<0.001 for both lines) (Fig. 5).

This information was used to infer that the susceptibility factor was present in the two deletion lines carrying the smaller deletions (del4DS-2 and del4DS-4) but was lost in the two lines containing the larger deletions (del4DS-3 and del4DS-1). Hence, the FHB susceptibility factor appears to reside between the deletion breakpoints of del4DS-4 and del4DS-3—a 31.73 Mbp interval (Fig. 5).

**Fig. 6.**
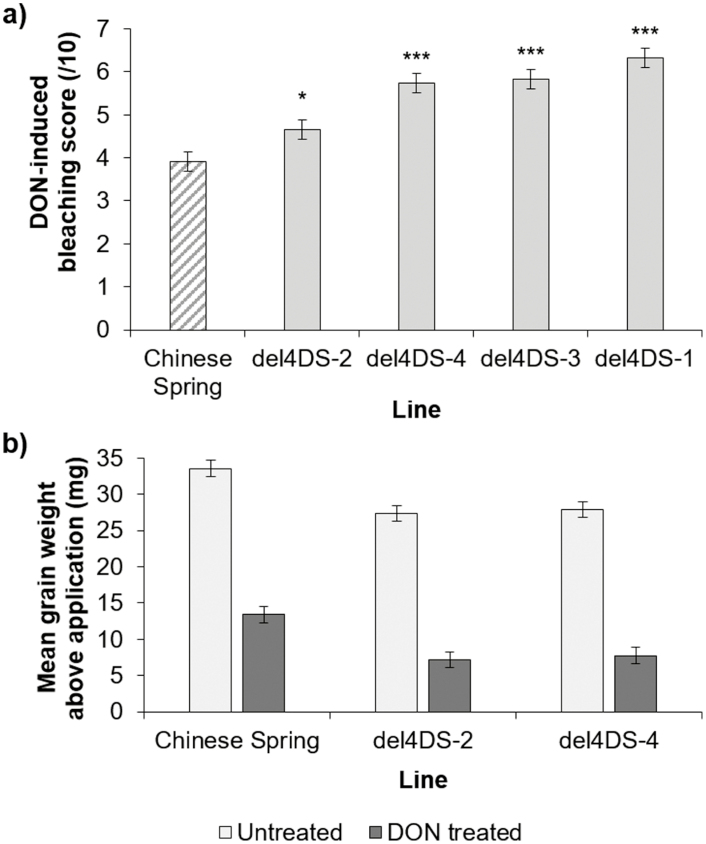
DON application experiment to heads of Chinese Spring and four terminal deletion lines. (a) Average DON bleaching scores at 8 d post-application. Predicted means were generated using a linear mixed model. Error bars are ±SE. *P*<0.001 compared with Chinese Spring. (b) DON-treated and untreated mean grain weights (mg) above the application point, or a comparable point in untreated heads, dissected after the experiment. Predicted means were generated using a linear mixed model. Error bars are ±SE.

The 4DS CSTD lines were also subjected to DON application to refine the position of the DON resistance factor also believed to be on 4DS (Fig. 6). Chinese Spring showed similar levels of bleaching to the previous experiment conducted on DT lines (mean=3.91). The CSTD line containing the smallest terminal deletion, del4DS-2, was significantly more susceptible to DON-induced bleaching than Chinese Spring (mean=4.66, *P*=0.019). However, del4DS-4 (mean=5.73, *P*<0.001) del4DS-3 (mean=5.83, *P*<0.001), and del4DS-1 (mean=6.32, *P*<0.001) all showed statistically significantly higher susceptibility to DON bleaching symptoms than Chinese Spring (Fig. 6).

Whilst all 4DS CSTD lines were significantly more susceptible to DON-induced bleaching than Chinese Spring, a greater differential was observed between del4DS-2 and del4DS-4 than was seen between Chinese Spring and del4DS-2. Grain were dissected from Chinese Spring, del4DS-2, and del4DS-4 to understand whether this result was also reflected in the grain data. Chinese Spring showed a 60.1% reduction in grain weight when comparing DON-treated and untreated grain. The 4DS CSTD lines, del4DS-2 and 4DS-4, showed similarly larger grain weight reductions as Chinese Spring (73.6% and 72.2%, respectively).

## Discussion

Previous studies have shown that barley is able to detoxify DON through glucosylation by the UGT HvUGT13248 (Schweiger *et al.*, 2010). This gene has been transgenically expressed in Arabidopsis where it was demonstrated to increase DON resistance (Shin *et al.*, 2012). Furthermore, expression of HvUGT13248 in wheat, under the maize ubiquitin promoter, increased FHB resistance, and transformants were demonstrated to more efficiently convert DON to the less toxic D3G (Li *et al.*, 2015). However, Xing *et al.* (2018) demonstrated that overexpression of a wheat UGT also increased FHB resistance and reduced the DON concentration in grain. How HvUGT13248 performs in wheat under its native barley promoter has not yet been demonstrated and hence the increase in resistance attributed to HvUGT13248 in wheat may be due to overexpression. HvUGT13248 is encoded by the gene HORVU5Hr1G047150 which is present near the centromere on chromosome 5H (Ensembl Plants). If the breakpoints in the wheat–barley 5HS and 5HL DT addition lines are not centromeric, this may explain the findings relating to the high level of resistance conferred by addition of both 5HS and 5HL. To confirm this, we designed primers specific to the barley copy of the UGT that does not amplify from the orthologous wheat copies in the wheat–barley additions. This assay confirmed that HvUGT13248 was present in the 5HL addition line and was absent in the 5HS addition line. Hence, it is likely that an independent source of FHB resistance is present on 5HS.

In this study, we also found that additions of barley chromosome 7H (7H add) and the short arm of chromosome 1H (1HS add), or the translocation of 1H to 2D (2D-1H trans), significantly increased Type II FHB resistance in the winter wheat variety Mv9kr1. Despite enhanced FHB resistance from the 7H addition to Mv9kr1, the addition of neither 7HS nor 7HL had an effect in the Asian spring wheat cultivar Chinese Spring. 1H addition lines were not available in the Chinese Spring–Betzes addition set, so this could not be compared between populations. These findings suggest that barley contains genes conferring Type II resistance that are lacking in one or both wheat varieties. The addition of barley chromosomes 5H and perhaps 1H and 7H is likely to offer the best opportunity of enhancing FHB resistance, when considering the use of wheat–barley introgressions.

We confirmed that the presence of the short arm of chromosome 4D was increasing Type II FHB susceptibility in three independent experiments. The loss of 4DS [line DT(4DL)] resulted in a high level of FHB resistance, whilst the loss of 4DL [line DT(4DS)] resulted in little change compared with Chinese Spring. Ma *et al.* (2006) phenotyped Chinese Spring DT lines for FHB susceptibility and they also reported an increase in FHB resistance in the line missing 4DS. Together, these studies strongly suggest that there is an FHB susceptibility factor in both winter (Mv9kr1) and spring (Chinese Spring) wheat genetic backgrounds. We applied purified DON to the 4D DT lines to test whether or not the susceptibility factor is being influenced by DON. However, the loss of 4DS resulted in increased DON susceptibility, assessed both by scoring DON-induced bleaching and by comparing grain weights. This would indicate that there is an independent DON resistance factor present on 4DS and that the susceptibility factor is increasing susceptibility to the fungus.

Endo and Gill (1996) developed a set of terminal deletion lines in Chinese Spring. The lines have deletions from the ends of each chromosome arm, varying in size. These stocks are a valuable resource for physically mapping genes to a defined interval of a chromosome arm. The lines were characterized using C-banding and the deletion size was breported as an FL value, effectively the proportion of the chromosome arm estimated to have been retained. C-banding is unlikely to be capable of reliably detecting more complex deletions, such as interstitial deletions or chromosome substitutions. Since their development, the Chinese Spring terminal deletion stocks have not been more precisely characterized using more recent advancements in genotyping. We have genotyped four lines containing terminal deletions of 4DS, using a total of 37 novel homoeologue non-specific markers spanning the chromosome arm. These markers take advantage of the hexaploid nature of wheat to create a robust genotyping assay for the detection of deletions on 4DS, and homoeologous regions on 4BS and 4AL. A similar assay was used by Chia *et al.* (2017) to verify deletions across homoeologous regions, but this study expands on this technique, using a much higher density of markers to characterize deletion size. Homoeologous genes are simultaneously amplified with a single pair of primers but are distinguishable due to differences in the size of PCR products corresponding to the A, B, and D genome copies. The signals from the retained homoeologues act as internal controls for a deletion in any homoeologue; in this case, the 4D copy. This technique verified that all four 4DS CSTD lines were indeed true terminal deletions and that the size of the deletions was consistent with the FL values calculated by Endo and Gill (1996). For the lines del4DS-2 and del4DS-4, the physical position of the deletion endpoint was determined to intervals <1 Mbp. The interval containing the deletion endpoint in del4DS-3 has been refined to ~2.3 Mbp. The breakpoint in the largest deletion, del4DS-1, was less precisely characterized (29.8 Mbp) because FHB susceptibility was associated with the interval between the deletion breakpoints in lines del4DS-4 and del4DS-3.

We performed FHB disease experiments on the four genotyped 4DS CSTD lines. This clearly demonstrated that the lines with the two smaller deletions (del4DS-2 and del4DS-4) retained the susceptibility factor and showed a similar phenotype to euploid Chinese Spring, while the two lines containing the larger deletions (del4DS-3 and del4DS-1) showed significantly increased FHB resistance and hence the susceptibility factor has presumably been lost. As del4DS-4 exhibited wild-type FHB susceptibility but del4DS-3 was comparatively more resistant, the cause must be situated between the deletion breakpoints of these two lines, restricting the susceptibility factor to a 31.7 Mbp interval containing 266 high confidence genes (Supplementary Table S1; IWGSC RefSeq v1.1). The positive effect from deleting the susceptibility factor appears to be restricted to 4D and hence it is possible that the gene responsible is 4D specific and does not possess homoeologues. BLAST searches of each 4D gene in the interval, followed by validation in Ensembl Plants, identified nine genes that appear to lack homoeologues and hence are 4D specific (Supplementary Table S1). Alternatively, the 4D homoeologue may be preferentially expressed or may have diverged in function compared with the 4A and 4B copies.

It may be considered surprising that an FHB susceptibility factor with such a powerful effect has not been detected before now. However, we hypothesize that the FHB susceptibility factor is highly conserved among wheat cultivars. The susceptibility factor exists both in the Hungarian winter wheat cultivar Martonvasari 9 and in the Asian spring wheat variety Chinese Spring. Preliminary experiments of a γ-irradiated Paragon line containing a deletion of the entire 31.7 Mbp FHB susceptibility interval indicates that this line possesses potent Type II resistance and hence confirms that the susceptibility factor is also present in the UK spring cultivar Paragon (data not shown). If there was sufficient allelic variation at the locus, the effect of the susceptibility factor is likely to have been detected as an FHB QTL in existing mapping populations. In the absence of such reports, we predict that the FHB susceptibility factor is fixed in both spring and winter wheats.

*Reduced height-D1* (*Rht-D1*) is located on the short arm of chromosome 4D. The mutant alleles *Rht-D1b* and homoeologous *Rht-B1b* (on chromosome 4B) result in reduced gibberellin sensitivity, causing decreased stem elongation and hence reduced plant height. These semi-dwarfing alleles were widely deployed during the Green Revolution and resulted in greatly increased crop yields (Hedden, 2003). However, *Rht-D1b* and *Rht-B1b* have also been implicated in increased susceptibility to FHB (Srinivasachary *et al.*, 2009; Saville *et al.*, 2012; Buerstmayr and Buerstmayr, 2016). Both Chinese Spring and Paragon possess the wild-type allele *Rht-D1a* (TraesCS4D02G040400) (P. Nicholson, personal communication). *Rht-D1* is at 18.78 Mbp on chromosome 4D, meaning that *Rht-D1* is distal to the FHB susceptibility interval identified in this study (EMBL-EBI, 2019). *Rht-D1* will have been deleted in the smallest terminal deletion line (del4DS-2), which did not show altered FHB symptoms. Furthermore, no obvious sign of altered plant height was observed in any of the Chinese Spring 4D DT or terminal deletion lines (data not shown). For these reasons, *Rht-D1* is unlikely to be responsible for the altered FHB susceptibility observed in the 4DS deletion lines.

We also used 4D CSTD lines in a DON application experiment. All four lines showed increased susceptibility to DON-induced bleaching compared with Chinese Spring. The effect on grain weight was less pronounced compared with the 4D DT lines. However, both del4DS-2 and del4DS-4 showed greater reductions in grain weight compared with Chinese Spring. These data suggest that DON resistance is associated with the region distal to 51.6 Mbp on 4DS in Chinese Spring. Further experiments are necessary to confirm whether the DON resistance is present in additional wheat varieties.

Genetic resistance to fungal diseases is critical to the protection of food crops such as wheat. The search for and incorporation of resistance factors is common practice in cereal breeding. However, identifying novel sources of resistance to FHB is challenging and time consuming. FHB resistance is quantitative, highly polygenic, and often environmentally labile. Few large-effect FHB QTLs have been identified. Attempts to clone the gene underlying the best-known source of FHB resistance, the *Fhb1* QTL, have been inconsistent and controversial (Ma *et al.*, 2017; Rawat *et al.*, 2017; Steiner *et al.*, 2017; Su *et al.*, 2017). Su *et al.* (2018) identified that the presence of a deletion at the 5' end of a histidine-rich calcium-binding gene (*TaHRC*) within the *Fhb1* locus was sufficient in identifying varieties carrying *Fhb1*. Su *et al.* (2019) have since reported that *Fhb1* possesses enhanced resistance due to the loss of function of *TaHRC* and that the wild-type allele is hence functioning as a susceptibility factor. Li *et al.* (2019) also identified that a deletion of the *TaHRC* gene was responsible for *Fhb1* resistance. However, in conflict with the findings of Su *et al.* (2019), their data suggest that this is due to a gain of function resulting from a different start codon positioned upstream to the original (Li *et al.*, 2019). Our data on the 3HS-3BL centric fusion line do not suggest that 3BS contains a susceptibility factor, as the line was either wild-type-like or more highly susceptible to the spread of FHB. This concurs with Ma *et al.* (2006), who reported that the Chinese Spring DT line missing 3BS [DT(3BL)] was more susceptible to FHB, which is not compatible with the hypothesis that FHB resistance results from *Fhb1* being a loss-of-function susceptibility factor. It remains possible that more than one gene is responsible for FHB resistance conferred by *Fhb1*.

There has been relatively little research into susceptibility factors in cereals and how they may be used in plant breeding. The barley *mildew resistance locus o* (*Mlo*) is one of the earliest and best characterized examples of how disruption of a susceptibility factor could be exploited to improve disease resistance; in this case, to powdery mildew caused by the biotrophic fungus *Blumeria graminis* f. sp. *hordei* (Jorgensen, 1992). This topic is covered in review articles by Acevedo-Garcia *et al.* (2014) and Kusch and Panstruga (2017).

R genes, usually nucleotide-binding site-leucine-rich repeat (NBS-LRR) genes, are typically used by plants to detect and respond to attack by biotrophic fungi. However, necrotrophic pathogens have evolved methods of exploiting such plant defences to aid infection. *Parastagonospora nodorum* and *Pyrenophora tritici-repentis* are necrotrophic pathogens of wheat that utilize this strategy. Susceptibility to these diseases operates in an inverse gene-for-gene interaction, in which a fungal necrotrophic effector is detected by a corresponding host sensitivity gene product (usually an NBS-LRR), triggering a hypersensitive response that results in necrosis that benefits the fungus (Faris *et al.*, 2010). Absence of either the necrotrophic effector or host sensitivity gene makes the interaction impossible and host resistance is maintained. There have been few reports of how NBS-LRRs are involved in interactions with *Fusarium* spp. However, Zhang *et al.* (2019) found that the expression of an LRR gene appeared to increase susceptibility to *F. graminearum* in soybean (*Glycine max*).

*Fusarium graminearum* leads a hemibiotrophic lifestyle whereby the hyphal front remains surrounded by living tissue but cell death is triggered soon after colonization (Brown *et al.*, 2010). Phytohormones play important roles in defence and there is considerable evidence to suggest that *F. graminearum* modifies phytohormone signalling for its own benefit. Disruption of ethylene signalling in wheat (Chen *et al.*, 2009) and brassinosteroid signalling in barley and *Brachypodium distachyon* (Goddard *et al.*, 2014) resulted in enhanced resistance to FHB infection, suggesting that the fungus is exploiting phytohormone signalling in order to aid infection. Expression of 9-lipoxygenases are also manipulated by *F. graminearum* in bread wheat and *Arabidopsis thaliana* and hence operate as susceptibility factors (Nalam *et al.*, 2015).

In the present study, we provide compelling evidence for FHB susceptibility associated with a 31.7 Mbp interval on the short arm of chromosome 4D. We have demonstrated that the removal of the susceptibility interval is sufficient to significantly improve Type II FHB resistance. A population possessing smaller deletions is required to further refine the position of the FHB susceptibility factor. We intend to utilize a γ-irradiated population of the UK spring wheat variety Paragon (Shaw *et al.*, 2013; Wheat Genetic Improvement Network, 2019) to improve the resolution for the physical mapping of the FHB susceptibility factor. Once this has been achieved, we will use the Cadenza TILLING population to validate the effect of mutations in individual gene candidates (Uauy *et al.*, 2009).

## Supplementary data

Table S1. Genes present in the defined FHB susceptibility interval, including summarized functional annotations.

Fig. S1. DON-treated wheat heads showing representative bleaching for the assigned bleaching scores (/10).

Fig. S2. Agarose gel image following PCR of Chinese Spring–Betzes 5HS and 5HL addition lines using primers targeting the barley UDP-glucosyltransferase gene HORVU5Hr1G047150.

Fig. S3. Example outputs of five multiplexed homoeologue non-specific markers used to genotype 4DS CSTD lines.

eraa226_suppl_Supplementary_Figures_and_Tables

## Abbreviations

CSTD: Chinese Spring terminal deletion
dpi: days post-inoculation
DON: deoxynivalenol
D3G: DON-3-*O*-glucoside
DT: ditelosomic
FHB: Fusarium head blight
FL: fraction length
QTL: quantitative trait locus
UGT: UDP-glucosyltransferase
