# Supplementary material for: Type II Fusarium head blight susceptibility conferred by a region on wheat chromosome 4D
Source: J Exp Bot. 2020 May 30;71(16):4703–14. doi: 10.1093/jxb/eraa226 (PMC7410183; doi:10.1093/jxb/eraa226)
Supplement: eraa226_suppl_Supplementary_Figures_and_Tables [file eraa226_suppl_supplementary_figures_and_tables.pdf]

Table 1 The 266 genes in the interval associated with increased FHB susceptibility, their physical start and end positions (bp), a summary of functional annotation information, and whether each gene is believed to be 4D specific (y/n). Functional annotation information was extracted from Ensembl Plants. Genes were assessed for 4D specificity using BLAST searches of genomic gene sequences, followed by validation of each candidate 4D specific gene in Ensembl Plants to check for predicted homoeologues.

Gene ID	Gene start (bp)	Gene end (bp)	Functional annotation	4D specific (y/n)
TraesCS4D02G079900	53862639	53864410	Oxoglutarate/iron-dependent dioxygenase	n
TraesCS4D02G080000	54052706	54055530	No annotation	n
TraesCS4D02G080100	54126623	54127674	Zinc finger, Dof-type	n
TraesCS4D02G080200	54261815	54266356	DNA-binding pseudobarrel domain superfamily, B3 DNA binding domain	n
TraesCS4D02G080300	54407216	54408847	Zinc finger, GRF-type	n
TraesCS4D02G080400	54474398	54476568	DNA-binding pseudobarrel domain superfamily, B3 DNA binding domain	n
TraesCS4D02G080500	54604350	54605788	DNA-binding pseudobarrel domain superfamily, B3 DNA binding domain	n
TraesCS4D02G080600	54620793	54622251	Protein of unknown function DUF1685	n
TraesCS4D02G080700	54678101	54684917	ATP-dependent (S)-NAD(P)H-hydrate dehydratase, Ribokinase-like	n
TraesCS4D02G080800	54787728	54802178	Exocyst complex component, Sec3	n
TraesCS4D02G080900	54805532	54807867	Ribosomal protein S27e, Zinc-binding ribosomal protein	n
TraesCS4D02G081000	54808771	54812009	Pre-mRNA-processing factor, Prp40, WW domain	n
TraesCS4D02G081100	55046790	55052311	ClpA/B family	n
TraesCS4D02G081200	55049422	55059562	Endonuclease III-like	n
TraesCS4D02G081300	55064251	55068537	Eukaryotic translation initiation factor 3 subunit B	n
TraesCS4D02G081400	55244698	55250083	Uncharacterised protein family UPF0454	n
TraesCS4D02G081500	55250298	55251359	GUN4-like	n
TraesCS4D02G081600	55279294	55281404	No annotation	n
TraesCS4D02G081700	55283971	55287557	Serine/threonine-protein kinase, leucine-rich repeat domains	n
TraesCS4D02G081800	55351008	55357103	PUA-like superfamily, ASCH domain	n
TraesCS4D02G081900	55357193	55362808	Short-chain dehydrogenase/reductase	n
TraesCS4D02G082000	55486685	55490064	Protein of unknown function, DUF936	n
TraesCS4D02G082100	55559831	55564851	ApaG domain	n
TraesCS4D02G082200	55567003	55567725	MIZU-KUSSEI 1-like	n

Table 1 (continued) The 266 genes in the interval associated with increased FHB susceptibility, their physical start and end positions (bp), a summary of functional annotation information, and whether each gene is believed to be 4D specific (y/n). Functional annotation information was extracted from Ensembl Plants. Genes were assessed for 4D specificity using BLAST searches of genomic gene sequences, followed by validation of each candidate 4D specific gene in Ensembl Plants to check for predicted homoeologues.

Gene ID	Gene start (bp)	Gene end (bp)	Functional annotation	4D specific (y/n)
TraesCS4D02G082300	55581025	55584910	BSD domain	N
TraesCS4D02G082400	55644462	55645280	No annotation	n
TraesCS4D02G082500	55963623	55965161	Aspartic peptidase A1 family, xylanase inhibitor	n
TraesCS4D02G082600	56025694	56030131	Homeobox-leucine zipper protein GLABRA2/ANL2/PDF2/ATML1-like	n
TraesCS4D02G082700	56706592	56709999	COBRA-like	n
TraesCS4D02G082800	56717029	56722549	ER membrane protein complex subunit 10	n
TraesCS4D02G083000	56743297	56745153	Glycosyltransferase family 92	n
TraesCS4D02G083100	56946795	56950730	Transmembrane protein DDB_G0292058-like	n
TraesCS4D02G083200	57069447	57075695	PB1, RWP-RK domains	n
TraesCS4D02G083300	57076214	57077374	Leucine-rich repeat, cysteine-containing subtype	n
TraesCS4D02G083400	57077396	57077758	No annotation	n
TraesCS4D02G083500	57222044	57223288	Calcineurin-like phosphoesterase domain, ApaH type	n
TraesCS4D02G083600	57471892	57474869	Protein of unknown function DUF842	n
TraesCS4D02G083700	57475090	57477121	Tetratricopeptide-like helical domain superfamily	n
TraesCS4D02G083800	57478397	57489288	Protein transport protein Sec23	n
TraesCS4D02G083900	57677192	57681625	Acetolactate synthase, small subunit	n
TraesCS4D02G084000	57835789	57838099	Serine-threonine/tyrosine-protein kinase-like, leucine-rich repeat domains	n
TraesCS4D02G084100	57878461	57879601	TRAF-like	n
TraesCS4D02G084200	58132334	58140734	Lupus La protein, nucleotide-binding alpha-beta plait domain	n
TraesCS4D02G084300	58144605	58146894	No annotation	n
TraesCS4D02G084400	58147777	58155663	Haemolysin A /rRNA methyltransferase TlyA	n
TraesCS4D02G084500	58267318	58267641	No annotation	y
TraesCS4D02G084600	58269253	58270644	ARGOS/ARL/OSR1	n
TraesCS4D02G084700	58273059	58280657	Ankyrin repeat-containing domain superfamily	n

Table 1 (continued) The 266 genes in the interval associated with increased FHB susceptibility, their physical start and end positions (bp), a summary of functional annotation information, and whether each gene is believed to be 4D specific (y/n). Functional annotation information was extracted from Ensembl Plants. Genes were assessed for 4D specificity using BLAST searches of genomic gene sequences, followed by validation of each candidate 4D specific gene in Ensembl Plants to check for predicted homoeologues.

Gene ID	Gene start (bp)	Gene end (bp)	Functional annotation	4D specific (y/n)
TraesCS4D02G084800	58387029	58400144	Peptidase S1C	n
TraesCS4D02G084900	58946127	58947568	F-box domain, kelch-type beta propeller	n
TraesCS4D02G085000	59412226	59414888	F-box domain, Kelch-type beta propeller	n
TraesCS4D02G085100	59622440	59625190	Domain of unknown function, DUF1624	n
TraesCS4D02G085200	59642929	59645962	START domain	n
TraesCS4D02G085300	60119850	60129277	Zinc finger, PHD-type	n
TraesCS4D02G085400	60133121	60134657	AP180 N-terminal homology (ANTH) domain	n
TraesCS4D02G085500	60167721	60170131	Glycoside hydrolase family 1	n
TraesCS4D02G085600	60175878	60178243	Glycoside hydrolase superfamily	n
TraesCS4D02G085700	60876664	60881604	GTP-binding protein EngA	n
TraesCS4D02G085800	61128713	61149270	ABC transporter, type 1	n
TraesCS4D02G085900	61150149	61164923	AP endonuclease 1	n
TraesCS4D02G086000	61585793	61593640	DNA polymerase III	n
TraesCS4D02G086100	61593741	61594886	Protein of unknown function DUF707	n
TraesCS4D02G086200	61605044	61605987	Alpha crystallin/Hsp20 domain	n
TraesCS4D02G086300	61851477	61852646	Homeobox domain	n
TraesCS4D02G086400	61956011	61958020	Chlorophyll A-B binding protein	n
TraesCS4D02G086500	61958328	61971184	No annotation	n
TraesCS4D02G086600	61989921	61991834	No annotation	n
TraesCS4D02G086700	62096216	62097196	Polysaccharide biosynthesis domain, IRX15/IRX15L/IGXM	n
TraesCS4D02G086800	62098207	62103294	Serine/threonine-protein kinase	n
TraesCS4D02G086900	62117595	62121775	Rieske-like [2Fe-2S] domain, NirD-type	n
TraesCS4D02G087000	62303659	62304525	DNA-binding domain superfamily, AP2/ERF domain	n
TraesCS4D02G087100	62457806	62469699	Glycosyl-hydrolase family 116, beta-glucosidase GBA2-type	n

Table 1 (continued) The 266 genes in the interval associated with increased FHB susceptibility, their physical start and end positions (bp), a summary of functional annotation information, and whether each gene is believed to be 4D specific (y/n). Functional annotation information was extracted from Ensembl Plants. Genes were assessed for 4D specificity using BLAST searches of genomic gene sequences, followed by validation of each candidate 4D specific gene in Ensembl Plants to check for predicted homoeologues.

Gene ID	Gene start (bp)	Gene end (bp)	Functional annotation	4D specific (y/n)
TraesCS4D02G087200	62479799	62483162	PPM-type phosphatase	n
TraesCS4D02G087300	62549510	62549965	Protein of unknown function, DUF538, At5g01610-like superfamily	n
TraesCS4D02G087400	62727949	62734054	Peptidase S10, serine carboxypeptidase, alpha/beta hydrolase fold	n
TraesCS4D02G087500	62744738	62745968	Protein of unknown function DUF241, plant	n
TraesCS4D02G087600	62759116	62779941	CCR4-NOT transcription complex subunit 11	n
TraesCS4D02G087700	62825477	62826949	Alpha/Beta hydrolase fold	n
TraesCS4D02G087800	62877199	62880498	Zinc finger, PHD-type	n
TraesCS4D02G087900	63043113	63047741	Proton-dependent oligopeptide transporter family, MFS transporter superfamily	n
TraesCS4D02G088000	63221516	63221980	VQ motif-containing protein 4/11/13/19/31/33	n
TraesCS4D02G088100	63230441	63234680	Glycerophosphodiester phosphodiesterase domain	n
TraesCS4D02G088200	63251469	63257122	Glycosyl transferase, family 1	n
TraesCS4D02G088300	63572418	63573832	Alpha/beta hydrolase fold	n
TraesCS4D02G088400	63584575	63590516	Serine/threonine-protein kinase, leucine-rich repeat domains	n
TraesCS4D02G088500	64185175	64191035	Serine/threonine-protein kinase, leucine-rich repeat domains	n
TraesCS4D02G088600	64219119	64219629	Papain-like cysteine peptidase superfamily	n
TraesCS4D02G088700	64348092	64348814	Leucine-rich repeat domain superfamily	n
TraesCS4D02G088800	64402276	64403619	No annotation	n
TraesCS4D02G088900	64536143	64541201	NB-ARC, leucine-rich repeat domains	n
TraesCS4D02G089000	64542625	64547434	Major sperm protein (MSP) domain, WD40 repeat, immunoglobulin-like fold	n
TraesCS4D02G089100	64603666	64606581	Serine/threonine-protein kinase	n
TraesCS4D02G089200	64615083	64617745	Serine/threonine-protein kinase	n
TraesCS4D02G089300	64706830	64719914	Major sperm protein (MSP), protein kinase domains, WD40 repeat	y
TraesCS4D02G089400	64724946	64730275	NB-ARC, leucine-rich repeat domains	n

Table 1 (continued) The 266 genes in the interval associated with increased FHB susceptibility, their physical start and end positions (bp), a summary of functional annotation information, and whether each gene is believed to be 4D specific (y/n). Functional annotation information was extracted from Ensembl Plants. Genes were assessed for 4D specificity using BLAST searches of genomic gene sequences, followed by validation of each candidate 4D specific gene in Ensembl Plants to check for predicted homoeologues.

Gene ID	Gene start (bp)	Gene end (bp)	Functional annotation	4D specific (y/n)
TraesCS4D02G089500	65069024	65070276	No annotation	n
TraesCS4D02G089600	65074144	65076366	Serine/threonine-protein kinase	n
TraesCS4D02G089800	65111408	65120476	NB-ARC, leucine-rich repeat domains	n
TraesCS4D02G089900	65120793	65127062	NB-ARC, protein kinase, leucine-rich repeat domains	n
TraesCS4D02G090100	65172412	65172906	Tapetum determinant 1	n
TraesCS4D02G090200	65173956	65175499	Domain of unknown function DUF1618	n
TraesCS4D02G090300	65176212	65181056	Glycosyl hydrolase family 100	n
TraesCS4D02G090400	65531283	65539548	E3 ubiquitin-protein ligase CHFR, cysteine rich domain with multizinc binding, zinc finger, RING-type	n
TraesCS4D02G090500	65576568	65578302	Transcription factor, TCP	n
TraesCS4D02G090600	65860440	65862170	Serpin domain	n
TraesCS4D02G090800	65934232	65936617	B3 DNA binding domain, REM family	n
TraesCS4D02G090900	65937066	65939493	Tetratricopeptide-like helical domain superfamily, pentatricopeptide repeat	n
TraesCS4D02G091000	65939637	65947808	Armadillo-like	n
TraesCS4D02G091100	65940148	65940408	No annotation	n
TraesCS4D02G091200	66286732	66296649	WD40 repeat	n
TraesCS4D02G091300	66300546	66306072	CSTF2 C-terminal domain superfamily, RNA recognition motif domain, RNA-binding domain superfamily	n
TraesCS4D02G091400	66315527	66322939	ATP-dependent RNA helicase, Ski2-like	n
TraesCS4D02G091500	66508426	66516165	GDP-fucose protein O-fucosyltransferase	n
TraesCS4D02G091600	66564304	66565045	No annotation	n
TraesCS4D02G091700	66584084	66587356	Potassium channel, voltage-dependent, EAG/ELK/ERG, cyclic nucleotide-binding domain	n
TraesCS4D02G091800	66778754	66779801	No annotation	n

Table 1 (continued) The 266 genes in the interval associated with increased FHB susceptibility, their physical start and end positions (bp), a summary of functional annotation information, and whether each gene is believed to be 4D specific (y/n). Functional annotation information was extracted from Ensembl Plants. Genes were assessed for 4D specificity using BLAST searches of genomic gene sequences, followed by validation of each candidate 4D specific gene in Ensembl Plants to check for predicted homoeologues.

Gene ID	Gene start (bp)	Gene end (bp)	Functional annotation	4D specific (y/n)
TraesCS4D02G091900	66934652	66935332	Late embryogenesis abundant protein, LEA_2 subgroup	n
TraesCS4D02G092000	67114226	67118748	ABC-2 type transporter	n
TraesCS4D02G092200	67238838	67239765	Late embryogenesis abundant protein, LEA_2 subgroup	n
TraesCS4D02G092300	67274217	67275167	Dirigent protein	n
TraesCS4D02G092400	67325629	67326189	Dirigent protein	n
TraesCS4D02G092500	67331802	67335734	F-box-like domain superfamily	n
TraesCS4D02G092700	67461111	67467357	Protein-tyrosine phosphatase	n
TraesCS4D02G092800	67790721	67801509	FANCM/Mph1-like, helicase superfamily	n
TraesCS4D02G092900	68053010	68054395	SANT/Myb domain, homeobox-like domain	n
TraesCS4D02G093000	68421890	68427970	Nonaspanin (TM9SF), MFS transporter superfamily	n
TraesCS4D02G093100	68467072	68485197	Major facilitator superfamily	n
TraesCS4D02G093200	68659603	68664622	Mlo-related protein	n
TraesCS4D02G093300	68708345	68712387	Fe-S cluster assembly domain superfamily	n
TraesCS4D02G093400	68757050	68757475	No annotation	y
TraesCS4D02G093500	68808693	68811146	Short-chain dehydrogenase/reductase SDR	n
TraesCS4D02G093600	69126886	69130154	Fatty acid hydroxylase	n
TraesCS4D02G093700	69212967	69213856	Protein of unknown function DUF688	n
TraesCS4D02G093800	69249045	69258155	Quinoprotein alcohol dehydrogenase-like superfamily, F-box domain, WD40 repeat	n
TraesCS4D02G093900	69396598	69428332	SANT/Myb, SWIRM, homeobox-like, winged helix-like DNA binding domains	n
TraesCS4D02G094000	69483751	69495734	Armadillo, C2 domain	n
TraesCS4D02G094100	69541973	69543835	Heavy metal-associated domain, HMA	n
TraesCS4D02G094200	69645773	69649022	Aspartic peptidase, xylanase inhibitor	n
TraesCS4D02G094300	69653506	69655682	Aspartic peptidase, xylanase inhibitor	n

Table 1 (continued) The 266 genes in the interval associated with increased FHB susceptibility, their physical start and end positions (bp), a summary of functional annotation information, and whether each gene is believed to be 4D specific (y/n). Functional annotation information was extracted from Ensembl Plants. Genes were assessed for 4D specificity using BLAST searches of genomic gene sequences, followed by validation of each candidate 4D specific gene in Ensembl Plants to check for predicted homoeologues.

Gene ID	Gene start (bp)	Gene end (bp)	Functional annotation	4D specific (y/n)
TraesCS4D02G094400	69677974	69680281	NAC domain	n
TraesCS4D02G094500	69846691	69850833	Aldehyde dehydrogenase, NAD(P)-dependent	n
TraesCS4D02G094600	70117722	70121045	S-adenosyl-L-methionine-dependent methyltransferase	n
TraesCS4D02G094700	70225177	70238546	DNA-directed DNA polymerase, family B	n
TraesCS4D02G094800	70337033	70337665	Tetratricopeptide-like helical domain superfamily	n
TraesCS4D02G094900	70342742	70343599	Tetratricopeptide-like helical domain superfamily	n
TraesCS4D02G095000	70351067	70359215	Ubiquitin carboxyl-terminal hydrolase 7, ICP0-binding domain, protein kinase, U box domains, Zinc finger, RING/FYVE/PHD-type	n
TraesCS4D02G095100	70359396	70361834	Nucleophile aminohydrolases, proteasome subunit beta 4	n
TraesCS4D02G095200	70499070	70500140	No annotation	n
TraesCS4D02G095300	70668816	70671084	BURP domain	n
TraesCS4D02G095400	70982700	70989065	NAD kinase	n
TraesCS4D02G095500	70998593	71000393	Oxoglutarate/iron-dependent dioxygenase, Isopenicillin N synthase-like	n
TraesCS4D02G095600	71291915	71295328	G-protein beta WD-40 repeat, WDR44/Dgr2	n
TraesCS4D02G095700	71419642	71420983	Zinc finger, GATA-type	n
TraesCS4D02G095800	71531706	71532257	Zinc finger, RING-type	n
TraesCS4D02G095900	71824231	71825912	Fatty acid hydroxylase	n
TraesCS4D02G096000	71827693	71835883	BRCT domain	n
TraesCS4D02G096100	72233939	72238152	Serine/threonine-protein kinase, UspA	n
TraesCS4D02G096200	72667347	72679003	Protein translocase subunit SecA	n
TraesCS4D02G096300	72814214	72814627	No annotation	n
TraesCS4D02G096400	72908162	72912139	Fungal lipase-like domain, Alpha/Beta hydrolase fold	n
TraesCS4D02G096500	73208464	73209696	No annotation	n
TraesCS4D02G096600	73219301	73224757	Cytochrome P450	n

Table 1 (continued) The 266 genes in the interval associated with increased FHB susceptibility, their physical start and end positions (bp), a summary of functional annotation information, and whether each gene is believed to be 4D specific (y/n). Functional annotation information was extracted from Ensembl Plants. Genes were assessed for 4D specificity using BLAST searches of genomic gene sequences, followed by validation of each candidate 4D specific gene in Ensembl Plants to check for predicted homoeologues.

Gene ID	Gene start (bp)	Gene end (bp)	Functional annotation	4D specific (y/n)
TraesCS4D02G096700	73348562	73367271	Tetratricopeptide, ankyrin repeats	n
TraesCS4D02G096800	73383347	73387542	Tetratricopeptide, ankyrin repeats	n
TraesCS4D02G096900	73965898	73967868	Leucine-rich repeat domain superfamily	y
TraesCS4D02G097000	73990733	73993653	F-box-like domain superfamily	n
TraesCS4D02G097100	73995368	73998086	F-box domain	n
TraesCS4D02G097200	74078066	74081055	Ribosomal protein L19, Translation protein SH3-like domain	n
TraesCS4D02G097300	74094854	74097031	Rubredoxin-like domain	n
TraesCS4D02G097400	74158600	74160169	Amino acid transporter, transmembrane domain	n
TraesCS4D02G097500	74348245	74348523	No annotation	n
TraesCS4D02G097600	74352157	74353215	No annotation	n
TraesCS4D02G097700	74355118	74356827	Leucine-rich repeat	n
TraesCS4D02G097800	74365369	74368036	Armadillo-type fold	n
TraesCS4D02G097900	74369510	74374873	Magnesium transporter NIPA	n
TraesCS4D02G098000	74605940	74606383	No annotation	n
TraesCS4D02G098100	74607942	74613341	B3 DNA binding domain	n
TraesCS4D02G098200	75044371	75051370	Protein of unknown function DUF616	n
TraesCS4D02G098300	75051512	75052908	No annotation	n
TraesCS4D02G098400	75228376	75232348	Disulphide isomerase	n
TraesCS4D02G098500	75263995	75272737	VHS, GAT domains, target of Myb protein 1	n
TraesCS4D02G098600	75388852	75390183	Aspartic peptidase A1 family, xylanase inhibitor	n
TraesCS4D02G098700	75500645	75509207	Leucine-rich repeat, F-box domains	n
TraesCS4D02G098800	75552745	75555188	Dynamin superfamily	n
TraesCS4D02G098900	75777040	75785075	Phosphoribulokinase/uridine kinase	n
TraesCS4D02G099000	75786290	75788897	No annotation	n

Table 1 (continued) The 266 genes in the interval associated with increased FHB susceptibility, their physical start and end positions (bp), a summary of functional annotation information, and whether each gene is believed to be 4D specific (y/n). Functional annotation information was extracted from Ensembl Plants. Genes were assessed for 4D specificity using BLAST searches of genomic gene sequences, followed by validation of each candidate 4D specific gene in Ensembl Plants to check for predicted homoeologues.

Gene ID	Gene start (bp)	Gene end (bp)	Functional annotation	4D specific (y/n)
TraesCS4D02G099100	76017896	76019693	FAD-binding domain	n
TraesCS4D02G099200	76022769	76025159	Nucleoside diphosphate kinase	n
TraesCS4D02G099300	76603700	76604587	NAC domain	n
TraesCS4D02G099400	76608798	76611905	F-box, leucine-rich repeat domains	n
TraesCS4D02G099500	76623427	76625071	Tetratricopeptide-like helical domain, pentatricopeptide repeat	n
TraesCS4D02G099600	76833594	76836331	Diacylglycerol glucosyltransferase	n
TraesCS4D02G099700	77016818	77018504	Tetratricopeptide-like helical domain, pentatricopeptide repeat	n
TraesCS4D02G099800	77025555	77028386	F-box, leucine-rich repeat domains	n
TraesCS4D02G099900	77041028	77042268	NAC domain	n
TraesCS4D02G100000	77120156	77121980	Histone deacetylase	n
TraesCS4D02G100100	77123671	77126282	F-box, leucine-rich repeat domains	n
TraesCS4D02G100200	77126757	77129349	Aspartic peptidase domain, xylanase inhibitor	n
TraesCS4D02G100300	77274740	77278489	Aspartic peptidase domain, xylanase inhibitor	n
TraesCS4D02G100400	77279700	77289755	F-box, leucine-rich repeat domains	y
TraesCS4D02G100500	77293544	77297377	F-box, leucine-rich repeat domains	n
TraesCS4D02G100600	77907770	77908495	F-box, leucine-rich repeat domains	n
TraesCS4D02G100700	78365010	78368288	F-box, leucine-rich repeat domains	n
TraesCS4D02G100800	78430837	78432003	Transcription termination factor, mitochondrial/chloroplastic	n
TraesCS4D02G100900	78731817	78732368	No annotation	y
TraesCS4D02G101000	78779604	78781584	Berberine/berberine-like, FAD-binding domain	n
TraesCS4D02G101100	78793792	78795012	No annotation	n
TraesCS4D02G101200	78835736	78836322	No annotation	n
TraesCS4D02G101400	79009424	79010202	Myb/SANT-like domain	n
TraesCS4D02G101500	79211788	79222841	LIM-domain binding protein/SEUSS	n

Table 1 (continued) The 266 genes in the interval associated with increased FHB susceptibility, their physical start and end positions (bp), a summary of functional annotation information, and whether each gene is believed to be 4D specific (y/n). Functional annotation information was extracted from Ensembl Plants. Genes were assessed for 4D specificity using BLAST searches of genomic gene sequences, followed by validation of each candidate 4D specific gene in Ensembl Plants to check for predicted homoeologues.

Gene ID	Gene start (bp)	Gene end (bp)	Functional annotation	4D specific (y/n)
TraesCS4D02G101600	79265253	79272716	Ribonuclease H superfamily	n
TraesCS4D02G101700	79700144	79708793	LIM-domain binding protein/SEUSS	n
TraesCS4D02G101800	80140858	80146784	Protein kinase domain	n
TraesCS4D02G101900	80145426	80146718	No annotation	n
TraesCS4D02G102000	80220933	80221864	Heavy metal-associated domain, HMA	n
TraesCS4D02G102100	80834783	80837529	Myb, homeobox-like domains	n
TraesCS4D02G102200	80881996	80886599	GDP-L-galactose/GDP-D-glucose phosphorylase	n
TraesCS4D02G102300	80972056	80983002	Phosphatidylinositol 3-/4-kinase	n
TraesCS4D02G102400	81323577	81331154	Pyridoxal phosphate-dependent transferase	n
TraesCS4D02G102500	81392507	81394669	TRAF-like, Zinc finger, RING/FYVE/PHD-type, SIAH-type	n
TraesCS4D02G102600	81664710	81666516	Haem peroxidase	n
TraesCS4D02G102700	81907398	81909866	Serine/threonine-protein kinase, legume lectin domain	n
TraesCS4D02G102800	81913568	81913969	No annotation	n
TraesCS4D02G102900	81917167	81917726	No annotation	n
TraesCS4D02G103000	81918232	81921969	Alcohol dehydrogenase	n
TraesCS4D02G103100	81971499	81974375	Alcohol dehydrogenase	n
TraesCS4D02G103200	81974843	81980179	No annotation	y
TraesCS4D02G103300	81984987	81987448	Alcohol dehydrogenase	n
TraesCS4D02G103400	82013042	82022962	NB-ARC, leucine-rich repeat domains	y
TraesCS4D02G103500	82288391	82294649	Tetratricopeptide-like helical domain, pentatricopeptide repeat	n
TraesCS4D02G103600	82291769	82295390	Peptidase S10, serine carboxypeptidase, alpha/beta hydrolase fold	n
TraesCS4D02G103700	82445067	82449145	Zinc finger, Sec23/Sec24-type, von Willebrand factor A-like domain	n
TraesCS4D02G103800	82459086	82463148	Papain-like cysteine peptidase superfamily	n
TraesCS4D02G103900	82496556	82497458	Dirigent protein	n

Table 1 (continued) The 266 genes in the interval associated with increased FHB susceptibility, their physical start and end positions (bp), a summary of functional annotation information, and whether each gene is believed to be 4D specific (y/n). Functional annotation information was extracted from Ensembl Plants. Genes were assessed for 4D specificity using BLAST searches of genomic gene sequences, followed by validation of each candidate 4D specific gene in Ensembl Plants to check for predicted homoeologues.

Gene ID	Gene start (bp)	Gene end (bp)	Functional annotation	4D specific (y/n)
TraesCS4D02G104000	82567068	82567595	Dirigent protein	n
TraesCS4D02G104100	82723737	82730645	Pyruvate kinase	n
TraesCS4D02G104200	82730421	82731619	DnaJ domain	n
TraesCS4D02G104300	82738104	82739053	Tapetum determinant 1	n
TraesCS4D02G104500	82819385	82820191	Tapetum determinant 1	n
TraesCS4D02G104600	82824481	82830774	Protein kinase, UbiB domains	n
TraesCS4D02G104700	82858191	82859262	Glycosyl transferase, family 31	y
TraesCS4D02G104800	82866172	82870011	MORF/ORRM1/DAG-like	n
TraesCS4D02G104900	83134211	83138863	Methylthioribose-1-phosphate isomerase	n
TraesCS4D02G105000	83138830	83155445	mRNA capping enzyme, tyrosine specific protein phosphatase	n
TraesCS4D02G105100	83275445	83280821	Basic-leucine zipper domain	n
TraesCS4D02G105200	83282445	83285125	DNA-directed RNA polymerase, RBP11-like	n
TraesCS4D02G105300	83852778	83873785	Lon, substrate-binding domain, PUA-like	n
TraesCS4D02G105400	84172744	84173782	TLDc domain	n
TraesCS4D02G105500	84269157	84275160	ATPase, AAA-type	n
TraesCS4D02G105600	84276749	84279790	Ribosomal protein L10e	n
TraesCS4D02G105700	84291890	84297256	AUX/IAA, PB1 domains	n
TraesCS4D02G105800	84301738	84303781	AUX/IAA, PB1 domains	n
TraesCS4D02G106000	84544401	84548094	Serine/threonine-protein kinase, regulator of chromosome condensation 1/beta-lactamase-inhibitor protein II	n
TraesCS4D02G106100	84601345	84604297	Serpin domain	n
TraesCS4D02G106200	84761190	84763178	Frigida-like	n
TraesCS4D02G106300	84776206	84779392	60S ribosomal protein L10P	n
TraesCS4D02G106400	84789277	84790464	Protein of unknown function DUF1677, Oryza sativa	n

Table 1 (continued) The 266 genes in the interval associated with increased FHB susceptibility, their physical start and end positions (bp), a summary of functional annotation information, and whether each gene is believed to be 4D specific (y/n). Functional annotation information was extracted from Ensembl Plants. Genes were assessed for 4D specificity using BLAST searches of genomic gene sequences, followed by validation of each candidate 4D specific gene in Ensembl Plants to check for predicted homoeologues.

Gene ID	Gene start (bp)	Gene end (bp)	Functional annotation	4D specific (y/n)
TraesCS4D02G106500	84792932	84794117	Protein of unknown function DUF1677, Oryza sativa	n
TraesCS4D02G106600	84795853	84797947	Leucine-rich repeat	n
TraesCS4D02G106700	84985600	84987918	Serine-threonine/tyrosine-protein kinase	n
TraesCS4D02G106800	85001956	85011084	Protein shortage in chiasmata 1-like	n
TraesCS4D02G106900	85165671	85175559	Serine/threonine-protein kinase, leucine-rich repeat domains	n
TraesCS4D02G107000	85258245	85264752	Serine/threonine-protein kinase, leucine-rich repeat domains	n
TraesCS4D02G107100	85335780	85342635	Forkhead-associated (FHA), PIN domains	n
TraesCS4D02G107200	85578813	85582953	Serine/threonine-protein kinase, EF-hand domain	n
TraesCS4D02G107300	85588200	85588928	Integrator complex subunit 3	n

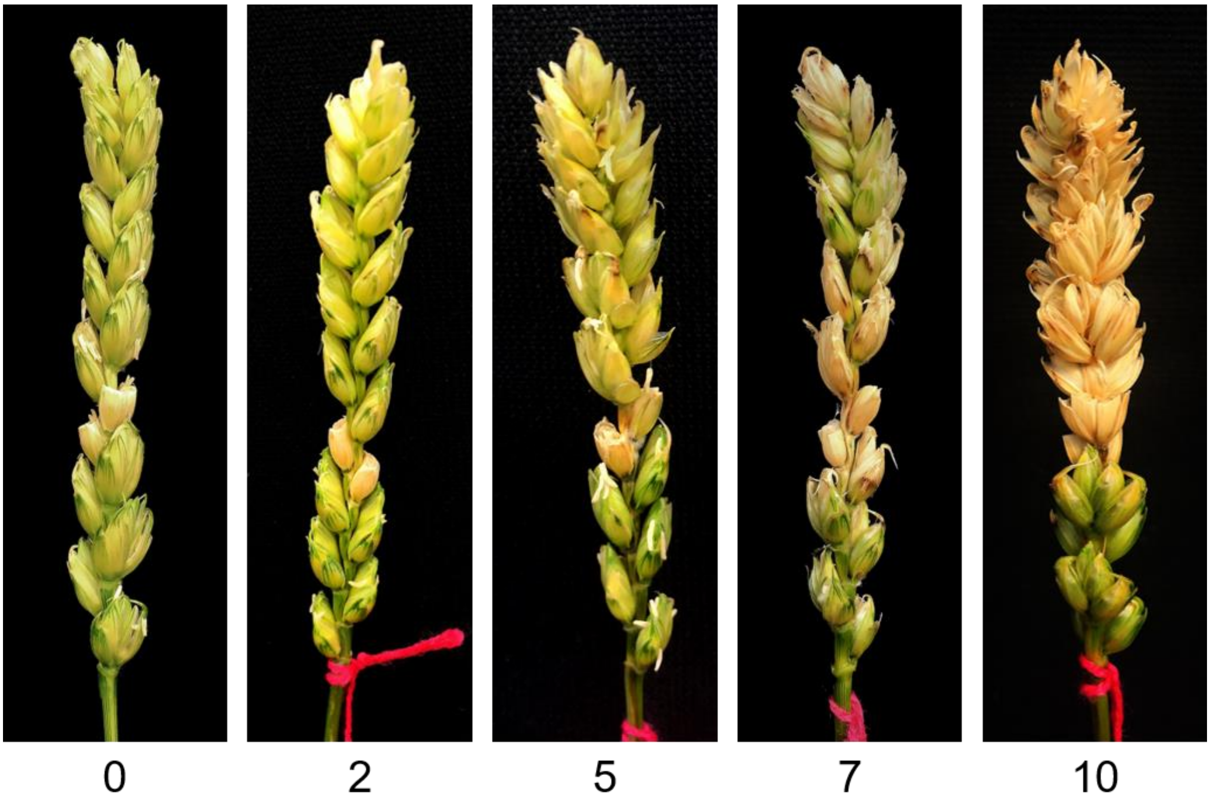

Figure S1 DON treated wheat heads showing representative bleaching for the assigned bleaching scores (/10).

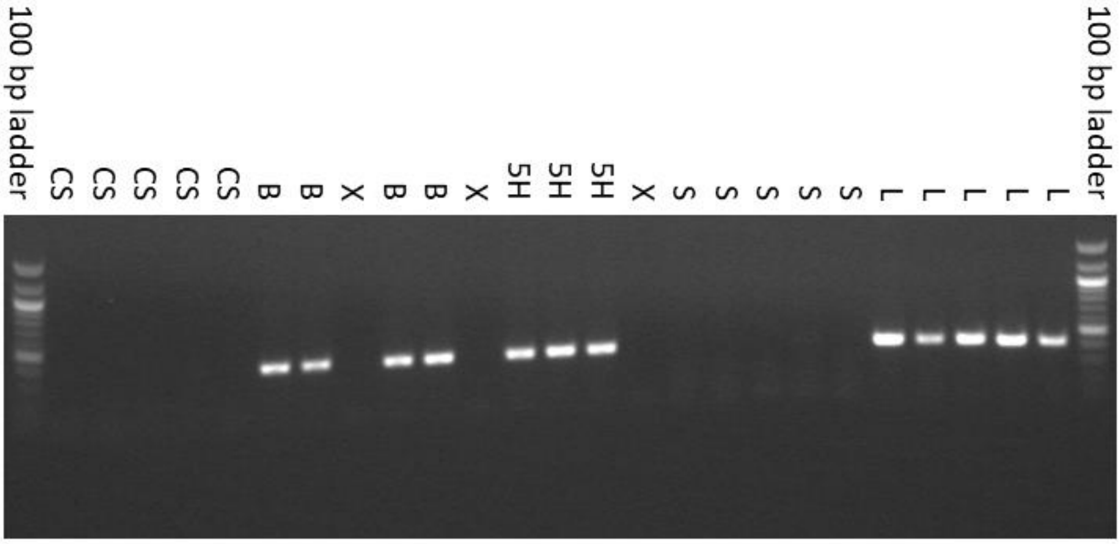

Figure S2 Agarose gel image following PCR using a marker targeting the barley UDP-glucosyltransferase gene, HORVU5Hr1G047150. Up to five DNA samples each of Chinese Spring (CS), Betzes (B), 5H addition line (5H), 5HS addition line (S) and 5HL addition line (L) were used, in addition to no template control samples (X).

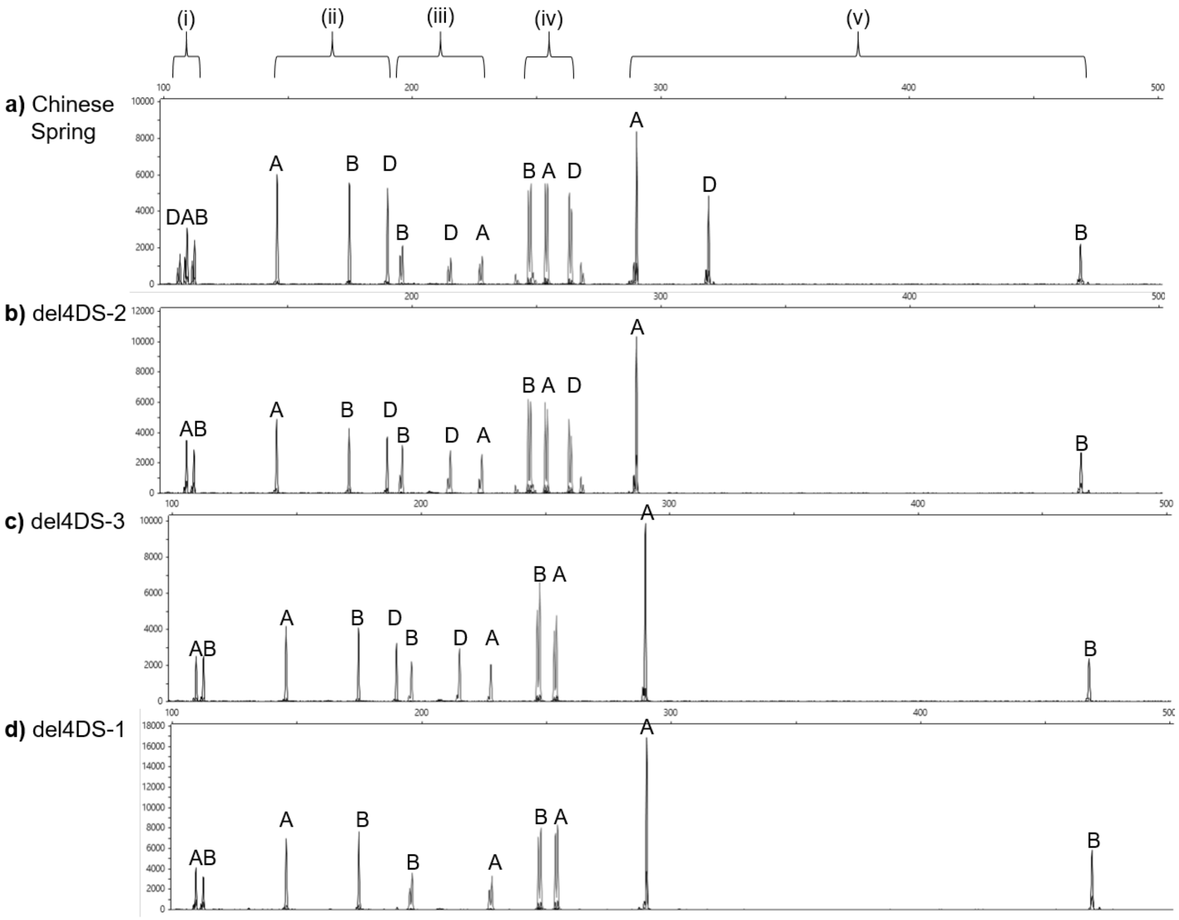

Figure S3 Example outputs of five multiplexed markers (i) BH0014, (ii) BH0030, (iii) BH0018, (iv) BH0017 and (v) BH0026, in a) Chinese Spring; b) del4DS-2; c) del4DS-3; d) del4DS-1. The line del4DS-4 showed the same deletion pattern as del4DS-2 for the markers visible in the selected multiplex and was hence omitted. X axis is fragment size (bp) and Y axis is the strength of fluorescence (relative fluorescence units). Images were extracted as screenshots from Peak Scanner 2 software (Applied Biosystems).
